# Preoperative knee cartilage degeneration in the patellofemoral joint results in worse clinical outcome scores 2 years after inverted V‐shaped high tibial osteotomy

**DOI:** 10.1002/jeo2.70690

**Published:** 2026-03-18

**Authors:** Yeonseok Jo, Dai Sato, Taku Ebata, Yoshiaki Hosokawa, Masato Hara, Koji Iwasaki, Masatake Matsuoka, Tomohiro Onodera, Eiji Kondo, Norimasa Iwasaki

**Affiliations:** ^1^ Department of Orthopedic Surgery, Graduate School of Medicine Hokkaido University Sapporo Japan; ^2^ Department of Functional Reconstruction for the Knee Joint, Graduate School of Medicine Hokkaido University Sapporo Japan; ^3^ Centre for Sports Medicine Hokkaido University Hospital Sapporo Japan

**Keywords:** inverted V‐shaped high tibial osteotomy, outcomes, patellofemoral cartilage, Whole‐Organ Magnetic Resonance Imaging Score (WORMS)

## Abstract

**Purpose:**

To determine whether preoperative whole‐organ knee magnetic resonance imaging findings are associated with 2‐year patient‐reported outcomes after inverted V‐shaped high tibial osteotomy (iV‐HTO). The hypothesis of this study was that preoperative tibiofemoral cartilage wear or meniscal lesion predicts worse outcomes 2 years after iV‐HTO, while patellofemoral joint degeneration does not have a significant impact.

**Methods:**

A retrospective review was conducted of iV‐HTO cases (2015–2022) with preoperative magnetic resonance imaging and 2‐year follow‐up data. Whole‐Organ Magnetic Resonance Imaging Score (WORMS) sub‐scores for cartilage, meniscus, bone marrow lesions, cysts and effusion were recorded for each compartment. Paired *t* tests were used to compare preoperative and post‐operative clinical and radiographic outcomes. Spearman correlations assessed associations between WORMS and Knee Injury and Osteoarthritis Outcome Score (KOOS) and Lysholm score. Multivariable linear regression analyzed predictors of 2‐year KOOS Symptoms, with patellar cartilage WORMS subscale (0–12) entered a priori and adjusted for preoperative symptoms, age, body mass index (BMI) and medial femoral condyle WORMS.

**Results:**

Forty‐six knees (39 patients; age, 55.5 ± 8.3 years; BMI, 27.9 ± 4.0 kg/m^2^) were included. The Lysholm score and KOOS improved significantly from baseline to 2 years. The post‐operative hip–knee–ankle angle, weight‐bearing line, femorotibial angle and medial proximal tibial angle differed significantly, while patellar height indices remained unchanged. Tilting angle and lateral shift ratio improved. There was no significant correlation between WORMS and Lysholm scores. WORMS cartilage subscale correlated negatively with KOOS Symptoms; strongest for the patella, followed by the medial femoral condyle. In the adjusted model, higher patellar WORMS independently predicted lower KOOS Symptoms at 2 years; other covariates were nonsignificant.

**Conclusions:**

iV‐HTO yields significant 2‐year clinical and radiographic improvements. However, advanced preoperative patellar cartilage degeneration independently predicted worse KOOS Symptoms despite coronal realignment and enhanced patellofemoral congruity. Preoperative patellofemoral cartilage grading should guide indications, counselling and preoperative planning.

**Level of Evidence:**

Level IV, retrospective cohort study.

**Abbreviations**
A‐P: anteroposterior
BMELL: bone marrow oedema‐like lesion
BMI: body mass index
CI: confidence interval
FsPD: fat‐suppressed proton‐density
FTA: femorotibial angle
HKA: hip–knee–ankle angle
HTO: high tibial osteotomy
iV‐HTO: inverted V‐shaped high tibial osteotomy
I–S: Insall–Salvati
KOOS: Knee Injury and Osteoarthritis Outcome Score
K–L: Kellgren–Lawrence
LCP: locking compression plate
LCW: lateral closing wedge
MCID: minimal clinically important difference
MFC: medial femoral condyle
MOW: medial opening wedge
MPTA: medial proximal tibial angle
MRI: magnetic resonance imaging
MTC: medial tibial condyle
NW: neutral wedge
OA: osteoarthritis
PF: patellofemoral
PF‐OA: patellofemoral osteoarthritis
PRO: patient‐reported outcome
TE: echo time
TF: tibiofemoral
TR: repetition time
TT‐TG: tibial tuberosity–trochlear groove
WORMS: Whole‐Organ Magnetic Resonance Imaging Score
%MA: weight‐bearing line percentage

## INTRODUCTION

High tibial osteotomy (HTO) is a widely established treatment for medial compartment osteoarthritis (OA) [20, 33]. While medial opening wedge (MOW) HTO is the most common technique, it has associated limitations, specifically the risk of inducing patella baja and increasing patellofemoral (PF) joint contact pressure due to distalization of the tibial tuberosity [18, 37]. Consequently, MOW‐HTO is often contraindicated for patients with concurrent PF joint OA [6, 40]. The inverted V‐shaped (iV) HTO is classified as a neutral wedge osteotomy, performed near the centre of rotation of angulation (CORA) [1, 21]. This unique geometry offers distinct advantages over standard MOW or lateral closing wedge (LCW) HTOs [24, 26]. Crucially, iV‐HTO prevents distalization of the tibial tuberosity, thereby preserving patellar height [22]. Furthermore, the procedure induces medialization of the patella, which theoretically reduces PF contact pressure and improves patellar tracking [12, 22]. Because of these biomechanical benefits, iV‐HTO fixed with a locking compression plate (LCP) has gained attention as an effective option for patients with severe varus deformity and those with PF joint pathology [34], cases where MOW‐HTO is considered a relative contraindication. However, despite these theoretical advantages, limited evidence exists regarding whether preoperative PF joint cartilage degeneration affects post‐operative clinical outcomes. Clarifying this relationship is essential to determine if iV‐HTO can be safely indicated for patients with pre‐existing PF OA.

The Whole‐Organ Magnetic Resonance Imaging Score (WORMS) is a validated semiquantitative method for evaluating structural abnormalities throughout the knee joint, including cartilage, marrow and menisci [16, 27, 38]. This system offers reproducible semiquantitative grading of cartilage integrity, meniscus status and bone marrow abnormalities across knee compartments on standard MRI, and it has demonstrated excellent intra‐ and inter‐reader reproducibility [27]. Previous studies have shown that detailed evaluation of meniscus, cartilage, and bone marrow lesions can help predict the progression of degenerative knee changes [8]. A comprehensive MRI‐based assessment of tissue‐specific structural joint health may help guide expectations for clinical outcomes.

This study aimed to evaluate the relationship between preoperative whole‐joint MRI findings and patient‐reported outcome measures after iV‐HTO. We hypothesized that while advanced cartilage degeneration or meniscal lesions in the medial tibiofemoral (TF) compartment would negatively impact post‐operative outcomes, preoperative PF joint degeneration would not significantly compromise clinical results, given the biomechanical advantages of iV‐HTO.

## METHODS

### Study design

A retrospective cohort study was performed, including consecutive patients who underwent iV‐HTO for medial OA at Hokkaido University Hospital between 2015 and 2022. Patients were identified from institutional surgical records, and written informed consent was obtained from all participants. The Institutional Review Board of Hokkaido University Hospital approved the study protocol (018‐0213). Eligible patients met the following criteria: (1) persistent knee pain due to medial OA that did not improve after three months of nonoperative therapy, (2) a knee that needed a wedge osteotomy angle >10°, (3) a loss of knee extension <10°, (4) range of knee motion >130°, (5) OA changes in the PF joint <Kellgren–Lawrence (K–L) classification grade 4 [19], (6) intact cruciate and collateral ligaments and (7) preoperative knee MRI available for review. Exclusion criteria were as follows: (1) prior ipsilateral knee surgery; (2) associated ipsilateral knee ligamentous injury requiring surgical treatment; (3) history of additional injury requiring surgery during the follow‐up window, except for plate removal; and (4) history of revision surgery and conversion to total knee arthroplasty. Two senior orthopaedic surgeons (K.E. and I.K.) with extensive procedural experience performed all operations. Each knee underwent radiological evaluation twice: preoperatively and 2 years post‐operatively. Each knee also underwent clinical evaluation at the same time.

### Arthroscopy and additional treatment

Diagnostic arthroscopy was routinely performed in all patients before the osteotomy to verify the indication and assess concomitant intra‐articular pathologies. The articular cartilage status of the medial and lateral TF compartments and the PF joint was evaluated and graded according to the International Cartilage Repair Society (ICRS) classification system. Concomitant procedures were performed when necessary to address chondral or meniscal injuries in the medial compartment.

### Surgical procedure of iV‐HTO

The surgical technique for iV‐HTO has been described previously (Figure 1a,b) [7]. During preoperative planning, the lateral wedge resection angle was selected to realign the mechanical axis so that it passed through a point located 65% lateral to the medial edge of the tibial plateau. Under C‐arm fluoroscopic guidance, a guidewire was inserted perpendicular to the anterior surface of the proximal tibia and advanced towards the planned apex of the iV osteotomy. The apex was positioned at the centre of the tibial condylar width and approximately 2.5 cm distal to the joint surface line. A wire insertion guide equipped with a protractor (Osferion Biomaterials Corp.), calibrated so that the sleeves matched the planned resection angle, was attached to the apex guidewire. Through these sleeves, two additional guidewires were inserted from the lateral side of the tibia, ensuring that each wire reached the apex guidewire with precision. A coronal ascending osteotomy was then created parallel to the anterior surface of the tibial tubercle using a thin oscillating saw, while preserving a 10‐mm proximal portion of the tibial tubercle. Lateral hemi‐wedge bone resection was performed along the previously placed guidewires using a thin oscillating saw and chisel. The anteromedial periosteum and a portion of the superficial medial collateral ligament were elevated along the medial osteotomy line. A Parallel Drill Guide (Osferion Biomaterials) was placed on the apex guidewire to align with the medial osteotomy line. Multiple parallel holes were drilled using a 2‐mm guidewire inserted through the sleeves. The medial osteotomy was completed along these holes using a thin chisel. Valgus correction of the tibia was achieved by creating an incomplete fracture at the apex of the V‐shaped osteotomy, followed by temporary fixation with two additional guidewires. Tibial fixation was performed with a lateral LCP plate (Osferion Biomaterials) installed on the lateral side. Morselized bone chips harvested from the lateral wedged resection were then implanted into the medial opening created after alignment correction (Figure 2a–f). Finally, a small locking plate was applied to the medial aspect of the osteotomy site. After irrigation, the subcutaneous tissues and skin were closed.

**Figure 1:**
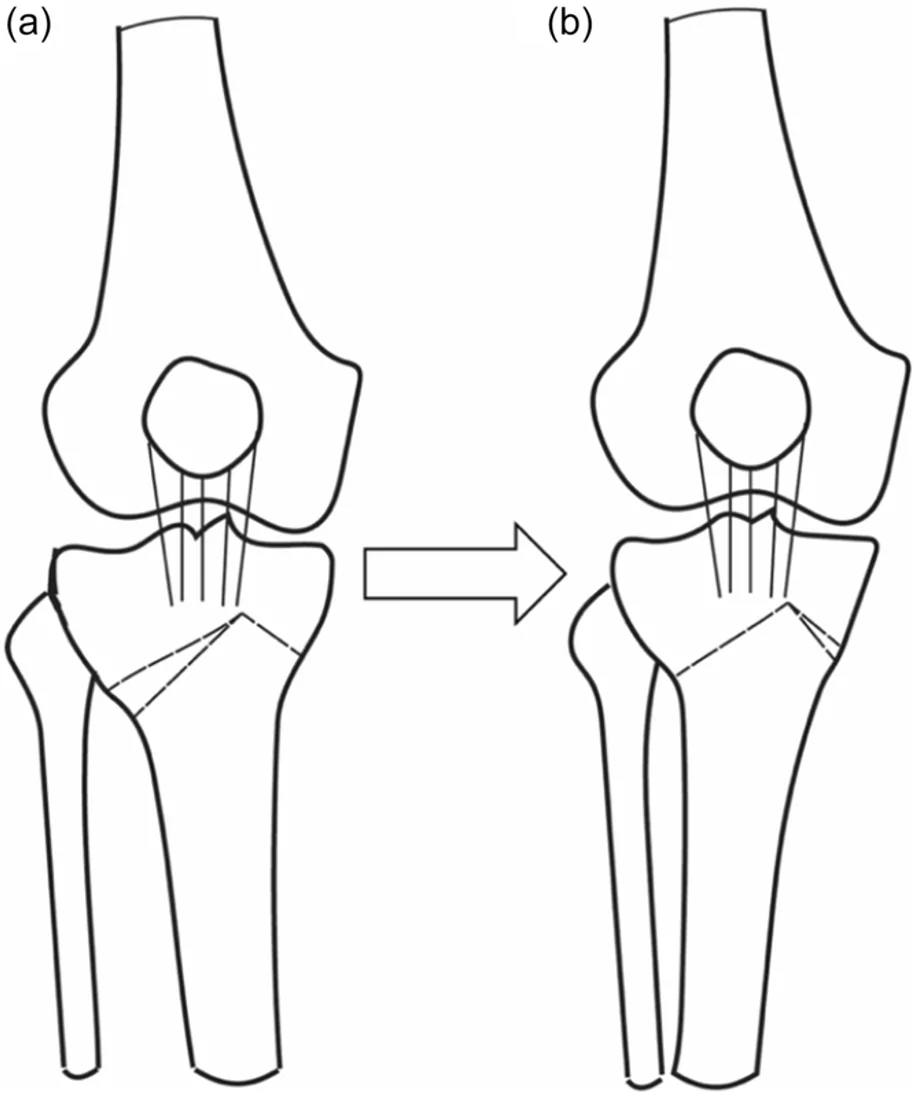
Diagrams of an inverted V‐shaped high tibial osteotomy: (a) a wedge of bone resected from the tibia beneath the lateral plateau in the shape of an inverted V. (b) Valgus correction and grafting of the resected wedge bone block into the triangular gap created beneath the medial plateau.

**Figure 2:**
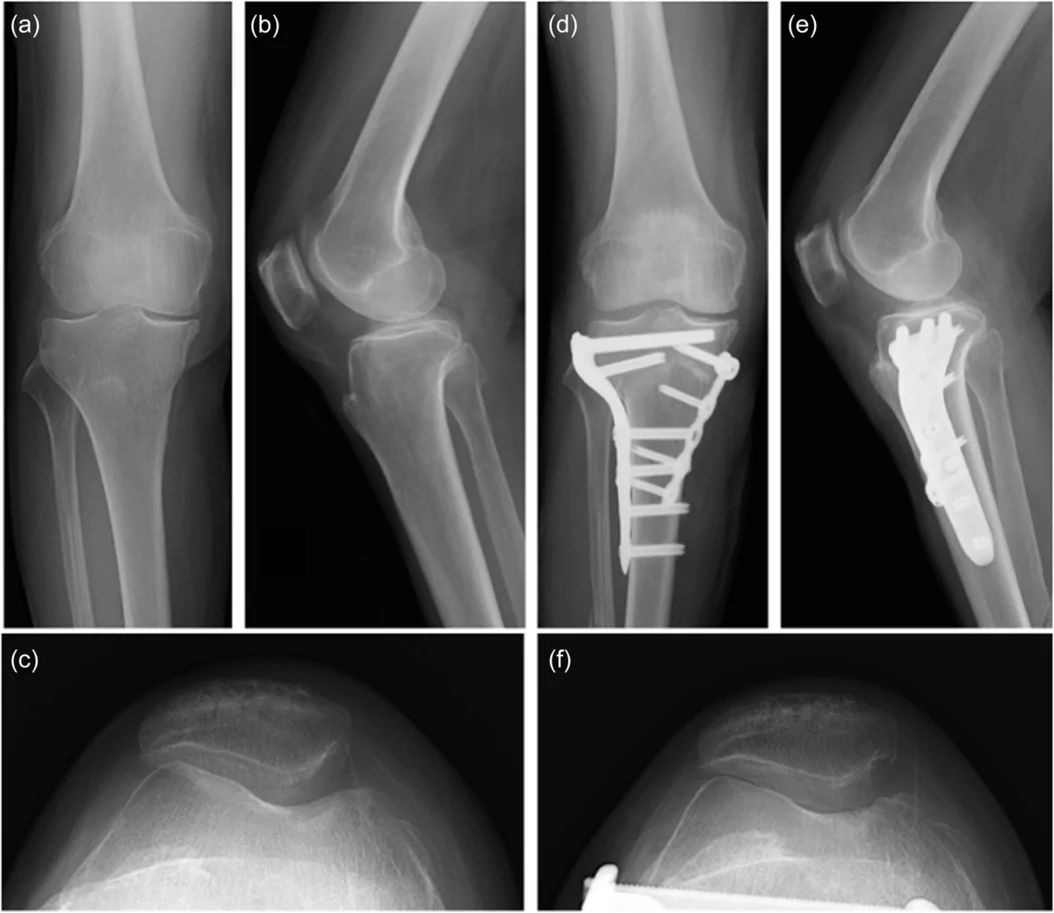
Pre‐ and post‐operative radiographs in a patient who underwent iV‐HTO. Before iV‐HTO: (a) anteroposterior, (b) lateral and (c) skyline view radiographs. After iV‐HTO: (d) anteroposterior, (e) lateral and (f) skyline view radiographs. iV‐HTO, inverted V‐shaped high tibial osteotomy.

### Post‐operative management

A previously reported rehabilitation protocol for iV‐HTO [21] was implemented post‐operatively. Quadriceps‐setting exercises and straight leg raises were permitted 1 day after surgery. Passive knee motion from 0° to 90° was allowed during the first 2 weeks, and active motion from 0° to 120° was encouraged thereafter. Partial weight bearing with crutches was allowed at 3 weeks, and full weight bearing was permitted at 5 weeks.

### Clinical evaluation/outcome scores

Overall clinical symptoms and knee function were evaluated in each patient using the Lysholm knee score [39] and the Knee Injury and Osteoarthritis Outcome Score (KOOS) [30], both preoperatively and at the 2‐year follow‐up.

### Radiological evaluation

The degree of OA at the femorotibial and PF joints was evaluated using the K–L classification [18]. Preoperative and post‐operative standing anteroposterior (A‐P) radiographs of the entire lower limb were taken to measure the hip–knee–ankle angle (HKA), the percentage of the mechanical axis crossing the joint line (%MA), the femorotibial angle (FTA) and the mechanical medial‐proximal tibial angle (MPTA). The %MA represents the horizontal distance from the mechanical axis to the medial edge of the tibial plateau divided by the width of the tibial plateau. Joint‐line obliquity (JLO) was measured on a long‐standing hip‐to‐ankle radiograph post‐operatively; it was defined as the angle between the line parallel to the floor and the tibial plateau, with a positive value of JLO meaning lateral inclination of the joint line [35]. Lateral radiographs were used to calculate the Insall–Salvati (I–S) [14] and Caton–Deschamps ratios [4]. The patellar tilt angle and lateral shift ratio [3, 15, 41] were measured on skyline radiographs with the knee flexed at 45°. The patellar tilt angle was defined as the angle between the line intersecting the widest bony structure of the patella and the line tangential to the anterior surface of the femoral condyle. Lateral shift was defined as the ratio of (1) the distance between the summit of the lateral femoral condyle and the point where two lines cross (the lateral edge of the patella and the line perpendicular to the summits of the femoral condyles) and (2) the distance between the summits of the medial and lateral femoral condyles of the femur.

### MRI semiquantitative grading

Preoperative knee MRI was acquired with a 1.5‐T whole‐body scanner (Achieva TX series; Philips Healthcare). The analyzed sequences included coronal, sagittal and axial T2‐weighted (field of view, 160; slice thickness, 3 mm; repetition [TR]/echo time [TE], 5000/80 ms) and coronal and sagittal fat‐suppressed proton‐density (FsPD)‐weighted images (field of view, 160; slice thickness, 3 mm; TR/TE, 3,500/9.2 ms). Two orthopaedic surgeons with >10 years of experience, blinded to subject characteristics, assessed all MRI scans. The modified WORMS is a semiquantitative scoring system that evaluates cartilage, bone marrow and meniscus abnormalities across six knee compartments and has demonstrated excellent reproducibility in multiple studies [10, 11, 17, 32, 36]. Each compartment and the entire knee had a tissue‐specific (e.g., cartilage and meniscus) maximum score in addition to a whole‐knee WORMS score. Summation WORMS scores were calculated for each lesion type for each examination: cartilage (0–84), meniscus (0–24), ligament and tendon (0–6), bone marrow oedema‐like lesions (BMELL, 0–18), subchondral cyst‐like lesions (0–18) and joint effusion (0–3). The osteophyte subscale was excluded because MRI has inherent limitations in assessing osteophytes [28].

### Inter‐ and intra‐reader reproducibility

Composite WORMS grading reproducibility was assessed in all patients. Two experienced orthopaedic surgeons (D.S. and Y.H.), blinded to all clinical data, independently performed all gradings to assess inter‐reader reproducibility. Intra‐rater consistency was determined by having one observer (D.S.) rescore the MRI 6 weeks after the initial assessment.

### Statistical analysis

The distribution of data was evaluated using the Shapiro–Wilk test. As the data generally followed a normal distribution, data are presented as mean ± standard deviation, and paired *t* tests were used to assess differences between preoperative and post‐operative clinical and radiological outcomes. Given the ordinal nature of the semi‐quantitative WORMS grading, Spearman rank correlation tests were used to assess associations between the WORMS scores and each clinical outcome. As an additional analysis, the association between post‐operative MPTA and post‐operative JLO was assessed using Pearson's correlation coefficient. Multivariable linear regression was used to identify independent predictors of post‐operative symptoms, with the KOOS Symptoms score 2 years after iV‐HTO as the dependent variable. To control for potential confounders, preoperative KOOS Symptoms, patellar cartilage WORMS score (0–12), medial femoral condyle WORMS score (0–12), age at surgery (years) and body mass index (BMI) (kg/m^2^) were entered a priori as covariates. Continuous variables were analyzed on their native scales, and linearity was checked using component‐plus‐residual plots. Results are presented as unstandardized coefficients (*β*) with 95% confidence intervals (CIs) and two‐sided *p* values; a negative *β* denotes worse KOOS Symptoms with higher predictor values. All statistical analyses were performed using GraphPad Prism version 10 (GraphPad Software). Inter‐tester reliability for categorical/ordinal MRI grading items (e.g., WORMS) was assessed using weighted Cohen's kappa (*κ*). According to Landis and Koch [23], *k* values are interpreted as follows: less than 0.00 indicates poor agreement; a value of 0.00–0.20, slight agreement; a value of 0.21–0.40, fair agreement; a value of 0.41–0.60, moderate agreement; a value of 0.61–0.80, substantial agreement; and a value of 0.81–1.00, almost perfect agreement. Statistical significance was defined as *p* < 0.05.

## RESULTS

A total of 47 patients (58 knees) were initially included in this study. Eight patients (10 knees) were subsequently excluded: five patients (6 knees) were lost to follow‐up, one patient (2 knees) did not undergo preoperative MRI, and two patients had prior anterior cruciate ligament reconstruction. Consequently, 39 patients (46 knees) were included in the final analysis (Figure 3), representing a follow‐up rate of 83.0%.

**Figure 3:**
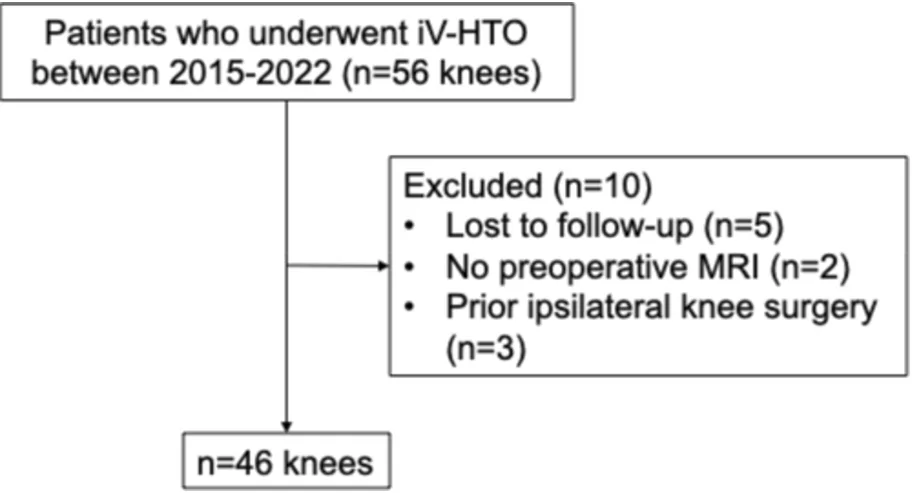
Study design. iV‐HTO, inverted V‐shaped osteotomy; MRI, magnetic resonance imaging.

### Patient demographics

The mean age of the patients was 55.5 years (range: 41–78 years). The mean BMI was 27.9 kg/cm^2^ (range 22.0–39.5). At the TF joint, the K–L OA grades were distributed as follows: Grade 1, 0 knees; Grade 2, 3 knees; Grade 3, 28 knees; and Grade 4, 15 knees. At the PF joint, grades were distributed as follows: Grade 1, 10 knees; Grade 2, 23 knees; Grade 3, 13 knees; and Grade 4, 0 knees (Table 1).

**Table 1:** Patient characteristics.^a^

Number of knees (patients)	46 (39)
Age (years)	55.5 ± 8.3
Male/female (patients)	29/17
Right/left (knees)	24/22
BMI (kg/m^2^)	27.9 ± 4.0
K–L grade (%)	
1	0 (0)
2	3 (6.5)
3	28 (60.8)
4	15 (32.7)
Patellofemoral OA (%)	
1	10 (21.7)
2	23 (50)
3	13 (28.3)
4	0 (0)
Arthroscopic surgery (%)	
Meniscectomy	5 (10.8)
Meniscus repair	3 (6.5)
Mosaicplasty	2 (4.3)

Abbreviations: BMI, body mass index; K–L, Kellgren–Lawrence; OA, osteoarthritis.

^a^ The data for age and BMI are presented as mean ± standard deviation.

### Arthroscopic evaluation

Arthroscopic assessment revealed that the medial compartment exhibited severe cartilage degeneration in all cases. In the PF joint, severe cartilage lesions (ICRS Grade 3 or 4) were observed in 19 knees (41.3%) in the trochlear groove and 17 knees (37.0%) in the patella (Table S1).

### Perioperative data and complications

The mean operative time was 131.2 ± 25.4 min. Regarding safety, there were no instances of intraoperative neurovascular injury or excessive blood loss requiring transfusion. However, three major complications occurred in this series (6.5%). One patient developed compartment syndrome post‐operatively, which was treated with emergent fasciotomy, resulting in a good recovery without permanent sequelae. Two cases of nonunion (pseudarthrosis) were identified; these were successfully managed by revision surgery with additional medial plating, and solid bone union was eventually achieved in both cases.

### Clinical outcomes

The Lysholm score significantly improved from the preoperative assessment to 2 years post‐operatively (*p* < 0.0001) (Table 2). The KOOS subscales similarly demonstrated significant improvement from the preoperative status to 2 years post‐operatively (Table 2). Importantly, these significant improvements in patient‐reported outcomes were observed in this large correction cohort (mean correction angle 13.4°).

**Table 2:** Clinical outcomes.^a^

	Preoperative	2 years post‐operative	*p*
Lysholm score	55.8 ± 12.9	87.3 ± 10.3	<0.0001
KOOS Symptom	60.8 ± 19.5	81.4 ± 12.6	<0.0001
KOOS Pain	53.4 ± 20.8	81.5 ± 12.1	<0.0001
KOOS ADL	66.6 ± 15.8	85.4 ± 11.5	<0.0001
KOOS Sports/Rec	31.9 ± 20.9	54.2 ± 21.4	<0.0001
KOOS QOL	34.7 ± 16.9	60.1 ± 21.6	<0.0001
KOOS total	55.7 ± 15.6	78.3 ± 11.3	<0.0001

*Note*: The *p* values were obtained from paired *t* tests comparing the pre‐ and post‐operative scores.

Abbreviations: KOOS, Knee Osteoarthritis and Injury Outcome Score; QOL, quality of life.

^a^ The data for clinical outcomes are presented as mean ± standard deviation.

### Radiological evaluation

The mean correction angle for iV‐HTO averaged 13.4 ± 3.63°, confirming that this cohort represents a large correction setting. Radiographic measurement results are presented in Table 3. Coronal lower leg alignment, HKA, %MA, FTA, MPTA and JLO showed significant changes from preoperative measurements to 2 years post‐operatively (*p* < 0.0001) (Table 3). The correlation between post‐operative MPTA and post‐operative JLO is shown in Figure S1. There were no significant differences in the Caton–Deschamps index and I–S ratio preoperatively or 2 years post‐operatively. PF joint congruity improved following iV‐HTO, with significant reductions in tilting angle and lateral shift ratio compared with preoperative values (*p* = 0.0074 and *p* = 0.011, respectively), indicating medialization of the patella (Table 3).

**Table 3:** Radiographic parameters.^a^

Radiographic parameter	Preoperative	2 years post‐operative	*p*
HKA	−9.2 ± 4.0	4.2 ± 0.96	<0.0001
%MA	8.0 ± 20.0	64.3 ± 10.8	<0.0001
FTA	183.3 ± 3.9	170.9 ± 3.5	<0.0001
MPTA	81.3 ± 3.8	93.8 ± 2.5	<0.0001
JLO	−1.6 ± 3.1	3.1 ± 2.4	<0.0001
Insall–Sarvati ratio	0.98 ± 0.15	0.99 ± 0.15	0.674
Caton–Deschamps index	0.85 ± 0.17	0.87 ± 0.15	0.570
Tilting angle	8.09 ± 4.09	5.56 ± 3.10	0.0074
Lateral shift ratio	0.14 ± 0.05	0.11 ± 0.13	0.011

*Note*: The *p* values are from paired *t* tests comparing pre‐ and post‐operative data.

Abbreviations: FTA, femorotibial angle; HKA, hip–knee–ankle angle; JLO, joint line obliquity; MPTA, medial proximal tibial angle; %MA, mechanical axis.

^a^ The data for radiographic outcomes are presented as mean ± standard deviation.

### Correlation of preoperative WORMS with post‐operative PRO scores

The preoperative total WORMS score averaged 31.3 ± 15.7 points (Table 4). The average WORMS subscale scores are presented in Table 4. Representative images of the patient with the worst WORMS are shown in Figure 4a,b. There was no significant correlation between WORMS and Lysholm scores. Significant correlations were identified between the WORMS cartilage subscale and the KOOS Symptoms and Stiffness subscales (*r* = −0.33; *p* = 0.023) (Table 5). Further analysis of correlations between WORMS cartilage scores in specific regions (patella and medial femoral condyle) and clinical scores revealed the following: Patella and KOOS Symptoms and Stiffness (*r* = −0.39; *p* = 0.0070) and medial femoral condyle and KOOS Symptoms and Stiffness (*r* = −0.34; *p* = 0.018) (Table 6).

**Table 4:** Preoperative MRI scores.^a^

WORMS	
Meniscus lesions (0–24)	3.1 ± 1.8
Cartilage lesions (0–84)	19.6 ± 11.3
Ligament abnormalities (0–6)	0.30 ± 0.50
Bone marrow (BMELL) (0–18)	5.1 ± 3.5
Subchondral cyst (0–18)	1.8 ± 1.8
Effusion (0–3)	1.2 ± 0.64
Total (0–153)	31.3 ± 15.7

Abbreviations: BMELL, bone marrow oedema‐like lesions; MRI, magnetic resonance imaging; WORMS, Whole Organ Magnetic Resonance Imaging Score.

^a^ The data for WORMS are reported as mean ± standard deviation unless otherwise indicated.

**Figure 4:**
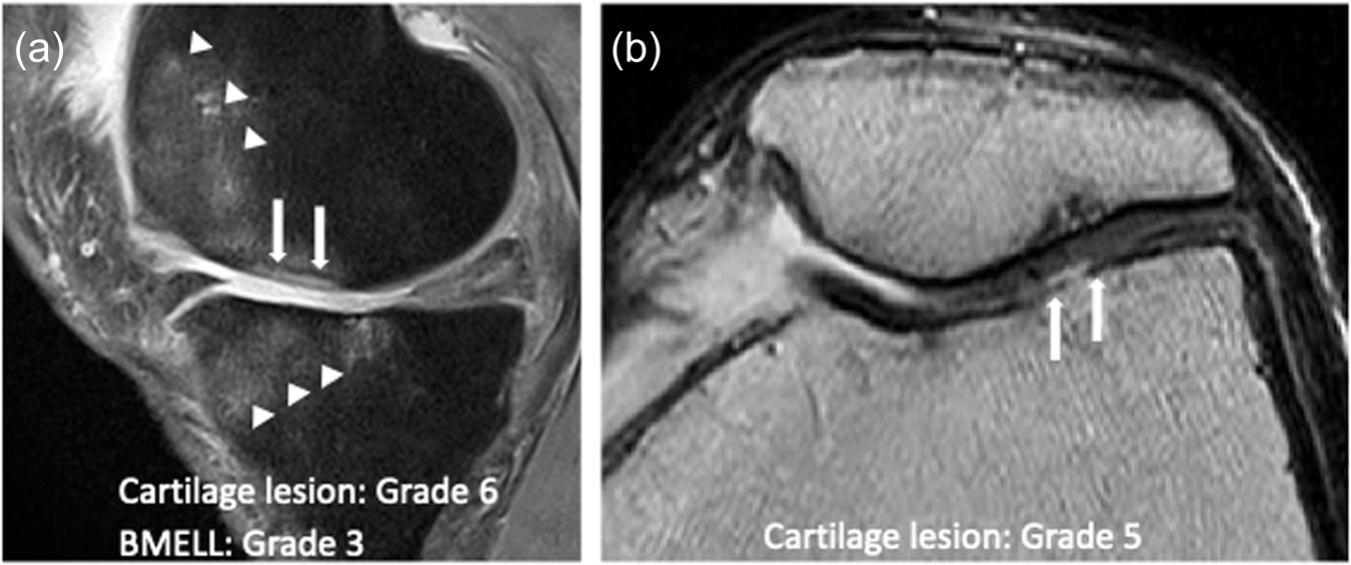
MRI findings in patients with high WORMS. (a) Sagittal FsPD‐weighted image shows a Grade 6 cartilage lesion (white arrows) in the MFC and a Grade 3 BMELL (white arrow heads) in the MFC and MTC. (b) Axial T2‐weighted image shows a Grade 5 cartilage lesion in the trochlea (white arrows). BMELL, bone marrow oedema‐like lesions; FsPD, fat‐suppressed proton‐density; MFC, medial femoral condyle; MRI, magnetic resonance imaging; MTC, medial tibial condyle; ORMS, Whole‐Organ Magnetic Resonance Imaging Score.

**Table 5:** Spearman correlations between the KOOS and WORMS.^a^

	KOOS Symptoms		KOOS Pain		KOOS ADL		KOOS Sport/rec		KOOS QOL	
	*r*	*p*	*r*	*p*	*r*	*p*	*r*	*p*	*r*	*p*
WORMS										
Meniscus lesions	−0.045	0.76	−0.022	0.88	0.085	0.57	0.083	0.58	0.022	0.88
Ligament abnormalities	−0.20	0.17	0.074	0.62	−0.091	0.54	−0.010	0.94	−0.15	0.31
Cartilage defects	−0.33	0.025	−0.066	0.66	−0.014	0.92	−0.073	0.62	−0.024	0.87
Bone marrow (BMELL)	−0.041	0.78	0.10	0.47	0.056	0.71	−0.0042	0.97	0.0030	0.98
Subchondral cyst	−0.15	0.29	0.070	0.64	0.089	0.55	0.084	0.57	−0.063	0.67
Effusion	0.042	0.77	−0.0053	0.97	−0.039	0.79	−0.059	0.69	−0.0088	0.95
Maximum score	−0.22	0.12	−0.014	0.92	0.015	0.91	−0.020	0.89	−0.031	0.83

Abbreviations: ADL, activities of daily living; KOOS, Knee Osteoarthritis and Injury Outcome Score; Sport/rec, sports and recreation; QOL, quality of life; WORMS, Whole‐Organ Magnetic Resonance Imaging Score.

^a^ Spearman's rank correlation (two‐sided). *r* = Spearman *ρ*; *p* = unadjusted *p* value. A negative *r* indicates that higher WORMS cartilage subscale scores were associated with lower KOOS scores (worse outcomes). *n* = 46 knees.

**Table 6:** Spearman correlations between the KOOS and WORMS cartilage sub‐scales.^a^

	KOOS Symptoms		KOOS Pain		KOOS ADL		KOOS Sport/rec		KOOS QOL	
	*r*	*p*	*r*	*p*	*r*	*p*	*r*	*p*	*r*	*p*
Cartilage sub‐scale										
Patella	−0.39	0.0070	−0.25	0.085	−0.11	0.46	−0.11	0.43	−0.16	0.26
Trochlea	−0.15	0.31	0.015	0.91	0.084	0.57	−0.034	0.82	0.039	0.79
Medial femoral condyle	−0.34	0.018	0.031	0.83	0.027	0.85	−0.0020	0.98	0.046	0.76
Lateral femoral condyle	−0.052	0.72	−0.079	0.60	−0.21	0.15	−0.17	0.23	−0.13	0.38
Medial tibial condyle	−0.21	0.15	−0.036	0.81	−0.013	0.93	−0.094	0.53	0.0049	0.97
Lateral tibial condyle	−0.28	0.057	−0.14	0.32	−0.18	0.23	−0.13	0.38	−0.16	0.28

Abbreviations: ADL, activities of daily living; KOOS, Knee Osteoarthritis and Injury Outcome Score; Sport/rec, sports and recreation; QOL, quality of life; WORMS, Whole‐Organ Magnetic Resonance Imaging Score.

^a^ Spearman's rank correlation (two‐sided). *r* = Spearman *ρ*; *p* = unadjusted *p* value. A negative *r* indicates that higher WORMS cartilage subscale scores were associated with lower KOOS scores (worse outcomes). *n* = 46 knees.

### Multivariable analysis

Multivariable linear regression demonstrated that patellar WORMS remained independently associated with worse 2‐year KOOS Symptoms (*β* = −1.52 points per 1‐point increase; 95% CI = − 2.93 to −0.10; *p* = 0.036) after adjustment for age, BMI, preoperative Symptoms score and medial femoral condyle WORMS (Table 7). Model fit: *R* ^2^ = 0.237; *F*(5, 40) = 2.48; *p* = 0.048. Other covariates were not statistically significant.

**Table 7:** Predictors of post‐operative KOOS Symptoms 2 years after iV‐HTO.^a^

Predictor	*β*	SE	95% CI	*p*
Patella WORMS (0–12)	−1.516	0.701	−2.932 to −0.100	0.036
Femur medial WORMS (0–12)	−0.712	0.469	−1.659 to 0.235	0.137
Preoperative KOOS Symptoms (points)	−0.125	0.101	−0.329 to 0.080	0.226
Age at surgery (years)	0.213	0.205	−0.201 to 0.627	0.304
BMI (kg/m^2^)	−0.240	0.437	−1.122 to 0.643	0.586

*Note*: Values are presented as ordinary least squares estimates (*β*) with SE, 95% CI and two‐sided *p* values (*α* = 0.05). A negative *β* indicates worse KOOS scores with higher WORMS scores. Model diagnostics: *n* = 46 knees; *R* ^2^ = 0.237; residual normality is not violated (D'Agostino–Pearson *p* = 0.056; Shapiro–Wilk *p* = 0.184); multicollinearity is low (all VIFs < 1.3).

Abbreviations: BMI, body mass index; CI, confidence interval; iV‐HTO, inverted V‐shaped high tibial osteotomy; KOOS, Knee Injury and Osteoarthritis Outcome Score; SE, standard error; WORMS, Whole‐Organ Magnetic Resonance Imaging Score; VIF, variance inflation factor.

^a^ Outcome: KOOS Symptoms at 2 years (0–100; higher = better). Predictors: preoperative KOOS Symptoms, WORMS cartilage subscale scores for the patella and medial femoral condyle (0–12; higher = worse), age (years) and BMI (kg/m^2^).

### Reproducibility of clinical readings

Weighted Cohen's kappa (*k*) values were calculated separately for each score. The inter‐reader agreement/k values for the meniscus, cartilage, ligament lesions, BMELL, subchondral bone cysts and effusion were 0.84, 0.86, 0.93, 0.93, 0.91 and 0.91, respectively. The intra‐reader agreement/k values for the meniscus, cartilage, ligament lesions, BMELL, subchondral bone cysts and effusion were 0.88, 0.92, 0.94, 0.95, 0.93 and 0.93, respectively. These findings demonstrate almost perfect inter‐ and intra‐reader agreement for WORMS.

## DISCUSSION

This procedure resulted in significant improvements in overall clinical and radiographic parameters at 2 years. PF cartilage degeneration, especially in the patella, was associated with poor clinical outcomes. Notwithstanding the improvement in radiological PF joint congruity, higher patellar cartilage WORMS were independently associated with lower KOOS Symptoms at 2 years in the adjusted model, whereas preoperative symptoms, age and BMI were not significant predictors.

Our data indicate that PF cartilage status, specifically the patellar WORMS cartilage subscale, is a key determinant of patient‐reported outcomes after iV‐HTO. Among the WORMS regions, the patella showed the strongest negative association with KOOS Symptoms, with a smaller correlation at the medial femoral condyle. In multivariable analysis adjusting for age, BMI and preoperative Symptoms, the patellar score remained an independent predictor of worse 2‐year KOOS Symptoms, whereas the other covariates were not significant. Even with substantial coronal realignment and improved PF congruity after iV‐HTO, as described in previous studies [21, 22] and confirmed in this study, advanced patellar cartilage degeneration appeared to limit symptomatic recovery.

iV‐HTO alters PF joint congruity [21, 22] because the hinge of the osteotomy is placed near the medial aspect of the tibial tuberosity, and the lateral osteotomy site is closed, allowing slight anteromedial advancement of the tibial tuberosity. This configuration tends to preserve patellar height and can reduce the tibial tuberosity–trochlear groove (TT‐TG) distance, thereby improving lateral shift and tilt. These findings were mirrored in our cohort by post‐operative decreases in these indices and the absence of clear PF deterioration. Nevertheless, our multivariate analysis identified the patellar WORMS cartilage subscore as an independent predictor of worse 2‐year KOOS Symptoms, even after appropriate coronal correction, indicating that iV‐HTO may optimize PF tracking but cannot fully compensate for advanced patellar cartilage loss. Historically, severe PF has been regarded as a contraindication for valgus‐producing HTO, and guidelines recommend avoiding osteotomy in the presence of relevant PF degeneration [6, 40]. Recently, related literature on lateral or hybrid closed‐wedge HTO has reported post‐operative clinical outcomes comparable to those of open‐wedge techniques despite more severe preoperative PF‐OA, suggesting that HTO can be compatible with PF disease [15, 25]. However, anticipated PF‐specific symptomatic gains have not been consistently verified. Taken together, these data imply that while iV‐HTO remains a reasonable option for varus OA knees with mild‐to‐moderate PF changes, substantial patellar cartilage degeneration tends to attenuate symptom improvement. Therefore, preoperative MRI grading of the patella should inform the indication and counselling with consideration of tempered expectations, adjunctive cartilage procedures, or tailored rehabilitation in patients with high patellar WORMS scores. Prospective studies with larger sample sizes are needed to confirm threshold effects and evaluate combined strategies for patients with substantial PF cartilage degeneration.

It is important to quantify how changes in patellar WORMS cartilage score translate into changes in patient‐reported outcomes. In this study, WORMS was used to characterize PF cartilage damage, and multivariate analysis identified the patellar WORMS cartilage score as an independent predictor of worse 2‐year KOOS Symptoms after iV‐HTO. Reported KOOS minimal clinically important difference (MCID) thresholds generally fall in the single‐ to low‐double‐digit range (8–15 points), although estimates vary by cohort, anchor and subscale [5, 13]. In our adjusted model, each 1‐point increase in the patellar cartilage sub‐score was associated with a 1.5‐point decrease in KOOS Symptoms; thus, a score of 6 would be expected to reduce KOOS Symptoms by ~9 points, near the lower bound of the commonly cited MCID range [5, 13]. This suggests potential clinical relevance, but interpretation should remain cautious because MCID values are context‐dependent and many cohorts report higher thresholds. Moreover, the KOOS primarily captures the overall knee status and is largely influenced by TF pathology; isolating the symptomatic impact of PF‐specific degeneration using a global instrument is inherently challenging [29, 39]. Notably, in WORMS, a patellar cartilage sub‐score of 6 typically denotes near‐full‐thickness loss [27], supporting the plausibility that advanced PF cartilage loss can blunt symptomatic improvement even with PF‐friendly iV‐HTO. Therefore, preoperative patellar assessment should inform indications, counselling, and preoperative planning, while confirmation in larger prospective cohorts is warranted.

Although MOWHTO is widely accepted as the standard and less technically demanding procedure, our cohort represents a large correction setting (mean correction angle 13.4°). In MOWHTO, increasing opening wedge size has been associated with a higher risk of lateral hinge fracture and delayed union/delayed gap healing [2, 9, 31]. Accordingly, inverted V‐shaped HTO was selected in this study as an indication‐specific strategy for large corrections, rather than to suggest superiority over MOWHTO. In particular, because our series required substantial correction (mean 13.4°), this indication differs from typical MOWHTO cohorts, and the present data demonstrate satisfactory short‐term outcomes even in this large‐correction population, even in this large‐correction population. However, notwithstanding the improvement in radiological PF joint congruity, preoperative patellar cartilage degeneration remained independently associated with worse post‐operative symptoms at 2 years, suggesting that iV‐HTO does not fully offset the impact of established PF degeneration in the short term. In addition, although post‐operative MPTA was relatively high, joint‐line obliquity assessed as JLO changed only modestly from preoperative to post‐operative assessments, and post‐operative MPTA was not significantly correlated with post‐operative JLO in our cohort (Figure S1). Future studies with longer follow‐up and comparative designs—including double‐level osteotomy—are needed to clarify the long‐term clinical relevance of post‐operative joint‐line orientation and to better define indications for iV‐HTO in large‐correction deformities.

This study has some limitations. First, PF‐specific scores, including the Kujala and KOOS‐PF [7], were not evaluated in our cohort. The changes in these scores were useful for understanding the effect of PF‐OA on the iV‐HTO procedure. Second, this study had a retrospective design and a small sample size. Third, this study did not evaluate the relationship between MRI findings and arthroscopic observations. Although arthroscopy provides a more sensitive and comprehensive assessment of the overall knee condition, MRI offers valuable preoperative information. Fourth, the average follow‐up period was only 2 years, and further analysis at longer follow‐up is needed. Since clinical decisions regarding osteotomy are made before surgery, understanding the joint condition using MRI is particularly important for predicting post‐operative outcomes.

## CONCLUSIONS

In patients with varus knee OA, iV‐HTO significantly improved the clinical outcomes and restored lower limb alignment. However, preoperative advanced patellar cartilage degeneration, quantified using the WORMS scoring system, may limit post‐operative clinical improvement. Therefore, careful preoperative MRI assessment of the PF joint is recommended to identify patients likely to experience less benefit from iV‐HTO alone and to guide appropriate surgical planning and patient counselling.

## AUTHOR CONTRIBUTIONS

Yeonseok Jo collected the data, made the analysis and drafted the work. Dai Sato conducted this study, supervised the data analysis and completed the draft. Yoshiaki Hosokawa and Masato Hara supported the data collection. Taku Ebata, Koji Iwasaki, Masatake Matsuoka, Tomohiro Onodera, Eiji Kondo and Norimasa Iwasaki interpreted the data. Eiji Kondo revised the draft critically.

## CONFLICT OF INTEREST STATEMENT

The authors declare no conflicts of interest.

## ETHICS STATEMENT

Ethical approval for this study was obtained from the institutional review board of Hokkaido University Hospital (approval no. 018‐0213). The requirement for informed consent was waived by the institutional review board because this study used anonymized retrospective data.

## Supporting information


**Supporting Information.** Supplementary file strobe checklist for this retrospective cohort study.

**Figure S1.** Scatter plot showing the correlation between postoperative medial proximal tibial angle (mpta) and postoperative joint‐line obliquity (jlo). Each dot represents one knee. Pearson correlation analysis showed no significant association (r = 0.24, p = 0.11).

T**able S1.** Distribution of ICRS grades in each compartment confirmed by arthroscopy.

## Data Availability

The data sets that support the findings of this study are available on request from the corresponding author. The data are not publicly available due to patient privacy regulations and institutional ethical restrictions.
