# Supplementary material for: Preoperative knee cartilage degeneration in the patellofemoral joint results in worse clinical outcome scores 2 years after inverted V‐shaped high tibial osteotomy
Source: J Exp Orthop. 2026 Mar 18;13(1):e70690. doi: 10.1002/jeo2.70690 (PMC13679884; doi:10.1002/jeo2.70690)
Supplement: Supplementary file 2 [file JEO2-13-e70690-s003.docx]

Supplemental Table 1. Distribution of ICRS grades in each compartment confirmed by arthroscopy^a^

|  | ICRS 0 | ICRS 1 | ICRS 2 | ICRS 3 | ICRS 4 |
| --- | --- | --- | --- | --- | --- |
| Patella, n (%) | 6 (13.1) | 14 (30.4) | 9 (19.6) | 15 (32.6) | 2 (4.3) |
| Trochlea, n (%) | 5 (10.8) | 9 (19.6) | 13 (28.3) | 16 (34.8) | 3 (6.5) |
| Medial femoral condyle, n (%) | 0 | 0 | 2 (4.3) | 17 (37.0) | 27 (58.7) |
| Lateral femoral condyle, n (%) | 18 (39.1) | 22 (47.8) | 6 (13.1) | 0 | 0 |
| Medial tibial condyle, n (%) | 0 | 1 (2.2) | 5 (10.8) | 15 (32.6) | 25 (54.4) |
| Lateral tibial condyle, n (%) | 17 (37.0) | 21 (45.6) | 7 (15.2) | 1 (2.2) | 0 |

^a^The data for ICRS grades are presented as number (%). ICRS, International Cartilage Repair Society
